# Combined Bentall, Coronary Artery Bypass Grafting and Implantation of Ascyrus Medical Dissection Stent Landed Inside a Thoracic Endovascular Aortic Repair Stent

**DOI:** 10.3390/jcm15093329

**Published:** 2026-04-27

**Authors:** Robert Grant, Pouya Nezafati, Bruce French

**Affiliations:** 1 Department of Cardiothoracic Surgery, Liverpool Hospital, Sydney, NSW 2170, Australia; robert.grant@health.nsw.gov.au (R.G.); bruce.french@health.nsw.gov.au (B.F.); 2 Department of Cardiothoracic Surgery, John Hunter Hospital, Newcastle, NSW 2305, Australia

**Keywords:** acute type A aortic dissection, AMDS, aortic root surgery, dissection stent

## Abstract

**Background:** Acute type A aortic dissection (ATAAD) is a life-threatening condition that may be complicated by malperfusion, particularly in patients with prior aortic interventions such as Thoracic Endovascular Aortic Repair (TEVAR). Management becomes increasingly complex when the dissection involves supra-aortic branches and compromises previously placed stents. **Methods:** We report the case of a 58-year-old male presenting with ATAAD and left lower limb paralysis, with a history of prior TEVAR. Imaging demonstrated an entry tear in the ascending aorta with extension into the distal left main and supra-aortic branches, resulting in a dissection flap obstructing the proximal end of the TEVAR stent. The patient underwent emergency surgical intervention including a Bentall procedure, coronary artery bypass grafting (CABG), and deployment of a small Ascyrus Medical Dissection Stent (AMDS) distally within the TEVAR stent. Pre-operatively, the patient had severe lower limb ischemia due to near-complete obstruction of distal flow. **Results:** Following surgical intervention, there was restoration of true lumen perfusion with resolution of malperfusion. The patient was successfully weaned from cardiopulmonary bypass, extubated on post-operative day 4, and discharged on day 7 with stable hemodynamics and intact bilateral lower limb perfusion. Post-operative computed tomography (CT) demonstrated a well-seated AMDS with no evidence of ongoing false lumen perfusion. At 30-day follow-up, there was no clinical or biochemical evidence of organ malperfusion. **Conclusions:** The use of an AMDS deployed within a pre-existing TEVAR stent may represent an effective strategy for managing complex ATAAD with malperfusion, particularly in cases requiring combined surgical interventions.

## 1. Introduction

Acute type A aortic dissection (ATAAD) is a high-risk condition requiring emergency surgery, with an incidence of 3–7 per 100,000 person-years and high early mortality [1]; despite advances in surgical techniques, operative mortality remains approximately 15–30% [2].

Often, the intimal entry tear is within the ascending aorta which extends into the aortic arch and to the descending aorta. In such cases, the dissection has most likely affected the supra-aortic branches for which total/hemi arch replacement has been known to be the surgical strategy. Conventional repair of the intimal flap has been reported to be associated with creation of new re-entries from false lumen which can further extend the dissection distally and cause collapse of the true lumen. The uncovered self-expanding endovascular stent, Ascyrus Medical Dissection Stent (AMDS), is introduced to prevent distal anastomotic new entry (DANE) by sealing the distal anastomosis and eliminating perfusion into the false lumen [3]. The stent is implanted into zone 0 of the aortic arch true lumen.

Short-term follow-up for the use of AMDS is reassuring [4]; however, reported outcomes of complex concomitant surgeries and AMDSs landed inside a previously implanted TEVAR valve are still scarce and need to be evaluated. Herein, we report a case of ATAAD with root involvement which underwent a concomitant Bentall and AMDS implantation into a TEVAR stent, to good effect.

## 2. Case Description

A 58-year-old male presented following a collapse and paralysis of left lower limb with signs of acute ischemic lower limb. He had a history of a TEVAR for a motor car accident traumatic aortic rupture 15 years previously. He was an ex-smoker on aspirin, and was otherwise functionally independent at baseline.

On admission he was also noted to have changes consistent with acute left coronary artery-related ischaemia. ATAAD was confirmed with a complete circumferential sinotubular junction (STJ) tear in a 63 mm diameter ascending aorta involving both coronary arteries. The Transesophageal Echocardiogram (TOE) revealed a severe aortic regurgitation. He was mildly tachycardic (110 bpm) with blood pressure at 130/60 mmHg, a urine output of 1 mL/kg/h, and a lactate of 2.8 mmol/L.

**Figure 1:**
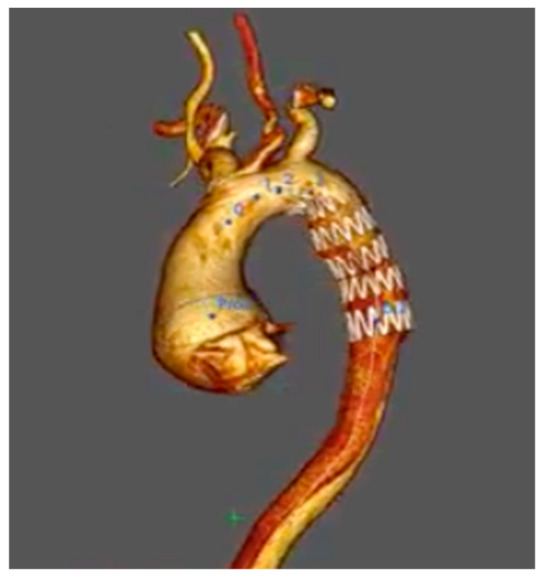
Pre-operative computed tomography (CT) aortogram. Figure legend: Pre-operative computed tomography aortogram demonstrating obstruction of the TEVAR lumen.

The redundant intimal flap had completely obstructed the TEVAR (Figure 1). The false lumen propagated across the aortic wall supported by the TEVAR and ended distally in the left common iliac artery. As a consequence of the obstructed TEVAR and very little flow within the false lumen beyond it, there was virtually no blood flow distal to the aortic arch. As noted, the lower limbs were severely acutely ischaemic.

Given the severe distal malperfusion and the intra-operative impression that the proximal TEVAR lumen was dynamically obstructed by the intimal flap, right femoral artery cannulation was chosen as the fastest route to establish cardiopulmonary bypass. Retrograde perfusion appeared to re-expand the true lumen across the TEVAR segment. Although additional imaging correlation was not available, the findings were most consistent with dynamic rather than fixed malperfusion.

The patient underwent surgical repair with a Bentall procedure, and the arch was stabilized with a small (40 mm–40 mm straight) AMDS deployed distally into the lumen of the TEVAR and proximally anastomosed to a 26 mm straight Dacron graft. Cardiopulmonary bypass (CPB) time was 407 min, with an aortic cross-clamp (ACC) time of 250 min. Deep hypothermia to 20 °C was employed. Cerebral protection was achieved using antegrade cerebral perfusion via the left carotid artery for 20 min, with a total duration of deep hypothermic circulatory arrest (DHCA) of 27 min.

**Figure 2:**
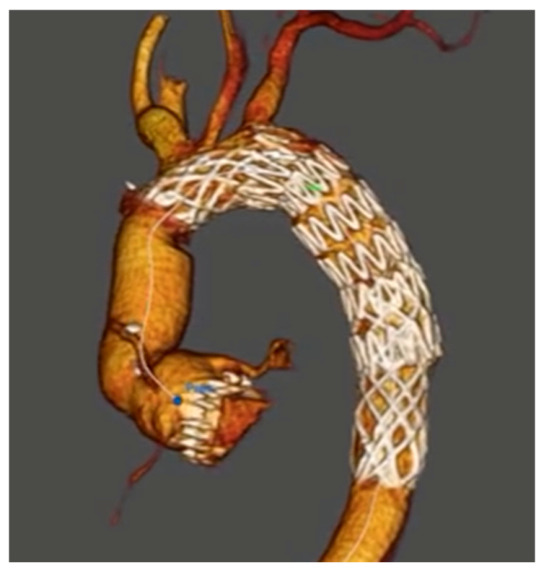
Post-operative computer tomography (CT) aortogram. Figure legend: Post-operative computed tomography aortogram showing well-seated AMDS and restored true lumen flow.

The right and left main coronary arteries were treated with BioGlue and re-attached to the root of a 25 mm Konect Aortic valve conduit graft with small external Teflon cuffs to support the anastomosis. On withdrawing the cardiopulmonary bypass (CPB), severe hypokinesia of the left anterior descending (LAD) and left circumflex (LCx) territories was noted, the decision was therefore made to graft the LAD and LCx using saphenous veins. The patient was successfully weaned from bypass and transferred to the intensive care unit. He was extubated on day 4 post-operatively with stable hemodynamics and without end organ damage including intact bilateral lower limb perfusion. The post-operative CT aortogram (Figure 2) revealed a well-seated AMDS.

The patient was ready for discharge on day 7 post-operatively to a rehabilitation center with no complications at the 30-day follow-up post-operation.

## 3. Discussion

Despite the advancement of pre-operative management and surgical technologies, morbidity and mortality of ATAAD are still considered to be high.

Hybrid approaches to aortic arch repair, including adjuncts such as the AMDS have been increasingly utilized to reduce distal anastomotic new entry and support favorable aortic remodeling [5]. Compared to more extensive arch replacement strategies, AMDS may offer a less invasive alternative with reduced operative complexity and shorter circulatory arrest times [6]. This is particularly relevant in complex ATAAD presentations, where balancing operative burden with effective distal aortic management remains a key consideration.

For complex cases special attention is required in decision making; in this case also for the management of lower limb malperfusion due to a blocked TEVAR, the management of coronary artery dissection, aortic root dissection with severe aortic regurgitation and the management of aortic arch dissection where a TEVAR has been previously implanted.

This case describes a rare use of AMDS deployed within a previously implanted TEVAR in the setting of ATAAD complicated by proximal TEVAR obstruction, with severe distal malperfusion, aortic root involvement, and coronary malperfusion requiring a concomitant Bentall procedure and CABG.

The distinguishing feature of this case lies not only in the presence of a prior TEVAR, but also in the proximal TEVAR obstruction caused by the dissection flap, resulting in near-complete loss of distal aortic flow, together with the need for a concomitant Bentall procedure and coronary artery bypass surgery in the same setting.

From a technical perspective, deployment of AMDS within a previously implanted TEVAR required precise alignment within the true lumen and careful integration with the distal anastomosis to avoid further luminal compromise.

This use of the AMDS successfully restored good end-organ perfusion. At the 30-day review, no signs of false lumen flow were evident on the post-operative CT of the chest at the aortic arch which reflects obliteration or thrombosis of the false lumen, radiologically. This describes the sealing promotion phenomena of the true lumen [7]. The use of AMDS is particularly advantageous in malperfusion when rapid true lumen expansion is required, and similar principles of true lumen expansion and false lumen decompression—demonstrated with TEVAR and bare-metal stenting (ASSIST)—may also contribute to improved aortic remodeling in this setting [8]. Moreover, there were no clinical neurovascular signs or symptoms of malperfusion. The concept of promoting true lumen expansion and reducing false lumen flow is also reflected in the PETTICOAT technique [9], which uses distal stent scaffolding to improve aortic remodeling. Although primarily described in type B dissection, its underlying principle is comparable to AMDS. In this case, AMDS provided proximal true lumen stabilization, achieving similar haemodynamic benefits without the need for additional distal endovascular intervention.

Concomitant treatment of ATAAD with total arch replacement and aortic root or CABG surgery increases the early post-operative mortality. However, Immohr et al. [10] revealed that combining root surgery and AMDS implantation is safe and does not impair the early post-operative outcome. Moreover, the cardiopulmonary bypass time and circulatory arrest time with AMDS is known to be shorter than the use of Thoraflex for arch replacement. This is an advantage in cases of complex concomitant surgeries such as this.

The follow-up period in this case is limited to 30 days, which precludes assessment of long-term aortic remodeling, durability of repair, and late complications. Longer-term surveillance with serial imaging is required to evaluate the sustained efficacy of AMDS in this setting.

## 4. Conclusions

Attention to decision making in complex ATAAD is of great importance. The clinical signs of re-perfusion from AMDS placement into a previously implanted TEVAR concurrent with a Bentall and CABG for Debaky I aortic dissection highlight another utility of AMDS which can further advance the standard of care for aortic dissections.

## 5. Learning Objectives

- Highlight a novel use of the Ascyrus Medical Dissection Stent in the setting of prior TEVAR.
- Demonstrate a surgical strategy for managing complex malperfusion in acute type A aortic dissection.
- Emphasize the role of hybrid techniques in restoring true lumen flow and end-organ perfusion.

## Data Availability

The original contributions presented in this study are included in the article. Further inquiries can be directed to the corresponding author.
